# Small dense low density lipoprotein-cholesterol and cholesterol ratios to predict arterial stiffness progression in normotensive subjects over a 5-year period

**DOI:** 10.1186/s12944-018-0671-2

**Published:** 2018-02-12

**Authors:** Gang Li, Hui-kun Wu, Xiao-wei Wu, Zhe Cao, Yuan-chao Tu, Yi Ma, Wei-qing Wang, Jian Cheng, Zi-hua Zhou

**Affiliations:** 1 Emergency Department, Hubei Provincial Hospital of Traditional Chinese Medicine, Wuhan, 430061 China; 2 Hubei Province Academy of Traditional Chinese Medicine, Wuhan, China; 3 Department of Hepatology, Hubei Provincial Hospital of Traditional Chinese Medicine, Wuhan, China; 4 Institute of Hepatology, Hubei Province Academy of Traditional Chinese Medicine, Wuhan, China; 5 Department of Thoracic Surgery, TongJi Hospital, TongJi Medical College, Huazhong University of Science and Technology, Wuhan, China; 6 Department of Cardiology, The Central Hospital of Wuhan, Wuhan, China; 7 Emergency Department, Wuhan General Hospital of Guangzhou Military Command, Wuhan, China; 8 Institute of Cardiology, Union Hospital, Tongji Medical College, Huazhong University of Science & Technology, Wuhan, China

**Keywords:** sdLDL-C, Cholesterol ratios, Arterial stiffness, Normotensive

## Abstract

**Background:**

Small dense low density lipoprotein-cholesterol (sdLDL-C), cholesterol ratios and carotid-femoral pulse wave velocity (cf-PWV) impart risk for all-cause morbidity and mortality independently of conventional cardiovascular disease (CVD) risk factors. This study was designed to identify feasible indicators for predicting arterial stiffness progression.

**Methods:**

We followed up 816 normotensive participants without diabetes or CVD for nearly 5.0 years. Cholesterol parameters, ratios and other clinical and laboratory data were collected at baseline. cf-PWV were measured at baseline and the end of follow-up.

**Results:**

PWV progression subjects had higher levels of PWV parameters, sdLDL-C and TG/HDL-C ratio. sdLDL-C and TG/HDL-C were significantly correlated with all PWV parameters. Multiple regression models showed that sdLDL-C was closely associated with follow-up PWV (β = 0.222, *p* < 0.001) and △PWV (β = 0.275, p < 0.001). TG/HDL**-**C was only one cholesterol ratios that associated with all PWV parameters. sdLDL-C (OR = 2.070, 95%CI: 1.162 to 3.688, *p* = 0.014) and TG/HDL-C (OR = 1.355, 95%CI: 1.136 to 1.617, *p* = 0.001) could significantly determine the progression of PWV after correction for covariates. High sd-LDL-C quantiles subjects were more likely to develop arterial stiffness progression than low quantiles (Tertiles 3 vs Tertiles1, RR = 2.867, 95%CI: 1.106 to 7.434, *p* = 0.03).

**Conclusion:**

We founded that sdLDL-C and TG/HDL-C ratio can independently predict arterial stiffness progression in normotensive subjects, and high level sdLDL-C and TG/HDL-C ratio were associated with a higher risk of arterial stiffness.

## Background

Arterial stiffness is aggravated with age, which is extensively associated with atherosclerotic vascular diseases. There are several ways of determining arterial stiffness, among which carotid-femoral pulse wave velocity (cf-PWV) is currently considered the gold standard [1]. cf-PWV directly reflects, and has the best clinical correlation to, aortic stiffness [1]. Epidemiological studies have confirmed that cf-PWV is a predictor of cardiovascular events and is one of the few indicators of arterial stiffness directly related to cardiovascular morbidity and mortality [2–4].

Several variables affect arterial stiffness, including age gender, blood pressure, high heart rate and life style, a variety of cardiovascular risk factors as well as genetic and systemic inflammation [5–8].Blood lipid parameters such as total cholesterol (TC), triglyceride (TG) and low-density lipoprotein cholesterol (LDL-C) are cardiovascular risk factors associated with atherosclerosis, but they cannot constantly predict arterial stiffness progression [9]. Recently, small dense low density lipoprotein-cholesterol (sdLDL-C) was regarded as one of the lipoprotein risk factors for coronary heart disease (CHD) and as the best marker of carotid atherosclerosis [10, 11]. However, little is known about the association between sdLDL-C and cf-PWV, especially in normotensive population. Cholesterol ratios are commonly used to reflect cardiovascular risk. A number of studies have shown that some of cholesterol ratios are associated with arteriosclerosis indicators such as carotid intima media thickness (CIMT) [12] and cardio-ankle vascular index (CAVI) [13]. In the Japanese diabetic population, high TG/HDL-C is independently associated with CAVI [14]. The TC/HDL-C ratio is highly correlated with other cardiovascular risk factors, including CIMT, high body mass index (BMI) and metabolic syndrome [12, 15]. However, the relationship between cholesterol ratios and cf-PWV, which is the gold standard to reflect arterial stiffness, has not been studied in a cohort with normal blood pressure. Additionally, whether or not these ratios can predict early arterial stiffness progression is unknown.

This study aimed to explore the relationship between sdLDL-C, cholesterol ratios and arterial stiffness progression which measured by the gold standard of cf-PWV. We followed up a normotensive population to identify an optimal indicator for predicting early arterial stiffness progression.

## Methods

### Study population

From September 2010 through March 2011, 1148 consecutive normotensive subjects without known cardiovascular disease according to WHO guidelines were selected for study. Subjects were invited to participate in a study for evaluation of cf-PWV. The inclusion criteria included normal blood pressure (systolic blood pressure [SBP] < 140 and diastolic blood pressure [DBP] < 90 mm Hg) and age of 18 years or higher. Patients with the following conditions were excluded: history of heart failure or cardiomyopathy, coronary heart disease, resting electrocardiogram (ECG) abnormalities (Q waves or left bundle branch block), cerebrovascular or peripheral artery disease and diabetes. Five years later, the subjects were invited to undergo the same arterial examinations. Overall, 816 subjects (71% of those invited) had their second visit from October 2015 through April 2016. Among the 332 subjects who did not have follow-up measurements, 198 lost contacts, 54 refused, and 20 did not return for unknown reasons. All participants provided written informed consent at the first visit.

In the present study, family income was classified as < 30,000, 30,000-100,000, and > 100,000 CNY/y. Educational level was categorized as low (no schooling, incomplete primary education, and primary education), middle (3 or 4 years of secondary education), and high (college and university education). Physical activity was classified into three groups using methods presented elsewhere [16]. We defined alcohol consumption as the weekly consumption of beer, wine and hard liquor. Current drinking was defined as alcohol consumption ≤8 ml per week according to the definition from the National Institute on Alcohol Abuse and Alcoholism [17]. We defined smokers as people who smoked at least one cigarette per day and continued for at least 1 year. We asked whether they currently smoked (Do you smoke currently?).

### Clinical investigations

Blood samples were collected in the morning after overnight fasting for at least 12 h. Blood was drawn into plastic tubes containing EDTA-2Na, and chilled in ice. Plasma was obtained by centrifugation at 4 °C, and kept at 0–4 °C until assays. All measurements were performed within 6 h. Fasting blood glucose(FBG), liver enzyme levels, serum lipid profiles, including TG, TC, LDL-C)and HDL-C were determined on a Olympus AU600 analyzer (Olympus, Tokyo, Japan). sdLDL-C was measured by the method of Hirano et al. [18] with minor modification [13] using the commercially available assay kit (sdLDL SEIKEN, Denka Seiken Co., Ltd., Tokyo, Japan). Apolipoproteins (apo) A-1, and B were measured by immunoturbidimetry (Daiichi Pure Chemicals Co., Ltd.,Tokyo). The ELISA technique was used to measure levels of high-sensitivity C reaction protein (hs-CRP, Immunodiagnostik Ag). The sensitivity of the assay was 0.05 ± 0.007. The cytokines interleukin-6 (IL-6) and tumor necrosis factor-α (TNF-á, R & D Systems Ltd.) were measured by ELISA in duplicates. The TNF-á and IL-6 assays detected concentrations down to 0.32 and 0.11 pg/ml, respectively. Intra-assay variability was < 9% for the 3 assays.

Supine blood pressure was assessed using a manual sphygmomanometer. After a 10 min rest period, blood pressure was measured 3 times and the average measurements used for statistical analyses.

cf-PWV measurements were obtained under the same conditions, including constant room temperature of 19 °C to 21 °C. We determined cf-PWV using a SphygmoCor automatic device (AtCor Medical, Sydney, Australia). SphygmoCor offers the possibility of cf-PWV measurements in two steps [19]. The first step is simultaneous recording of carotid pulse wave and ECG, and the second is recording of the femoral pulse wave and ECG. ECG recording during measurements is necessary for synchronization of carotid and femoral pulse wave times. Transit time between carotid and femoral pressure waves was calculated using the foot-to-foot method. The foot of the wave was identified using intersecting tangent algorithms. An abnormal cf-PWV was defined as standard cf-PWV that is superior to the decade-specific 90th percentile of age for normal subjects.

### Statistical analysis

The statistical software package SPSS18.0 (SPSS Inc., Chicago, IL, USA) was employed. Continuous variables in normal distribution were compared using Student’s t-test and analysis of variance (ANOVA). Categorical variables were analyzed using the chi-squared test. Patients were divided into 2 groups according to their gender and hypertension or cf-PWV progression. In this study, we defined cf-PWV > 12 m/s as a clinically significant outlier [20]. Subject with baseline cf-PWV < 10 m/s and follow-up cf-PWV > 12 m/s was regarded as PWV progression. In order to assess the difference between baseline PWV, follow-up PWV and△PWV (change from baseline PWV to follow-up PWV) with hypertension and PWV progression, we used generalized linear model (GLM) after adjusting for covariates. Partial Spearman correlation coefficients were used to clarify the association between PWV parameters and cholesterol components or ratios. To assess the independent value of sdLDL-C and different cholesterol ratios in predicting the progression of arterial stiffness, we used multivariate linear regression analysis models. We used the multivariate Cox regression model to analyze the predictive value of these ratios and sdLDL-C for cf-PWV progression. Cox proportional hazards model was used to evaluate the relative risk of progression of arteriosclerosis between sdLDL-C and TG/HDL-C three quantile subgroups. The data are expressed as the mean ± SD. *p* values < 0.05 were considered statistically significant.

## Results

A total of 816 consecutive subjects that met the inclusion criteria were enrolled and completed follow-up. The participant population included 476 (58.3%) men, and the average age of participants was 39.1 ± 7.8 years. Baseline blood pressure parameters were as follows: SBP (125.9 ± 9.4 mmHg), DBP (75.2 ± 7.1 mmHg), pulse pressure (PP) (50.7 ± 9.5 mmHg) and MAP (92.1 ± 6.5 mm Hg). Baseline mean cf-PWV was 9.90 ± 0.84 m/s and follow-up was 10.51 ± 1.12 m/s. The annualized average progression of cf-PWV was 0.12 ± 0.08 m/s/year. After 5 years of follow-up, 129 normal blood pressure subjects developed hypertension. Among them, grade Ihypertensive patients were 82, grade II were 38 and grade III were 9. In addition, 60 subjects used drugs to control blood pressure. At the end of follow-up, the cf-PWV of hypertensions were significantly higher than normal subjects (11.17 ± 1.43 vs 10.46 ± 1.10 m/s, *p* < 0.001). At the end of the follow-up, there were a total of 144 patients with cf-PWV progression, with an average of 12.70 ± 0.64 m/s.

**Fig. 1:**
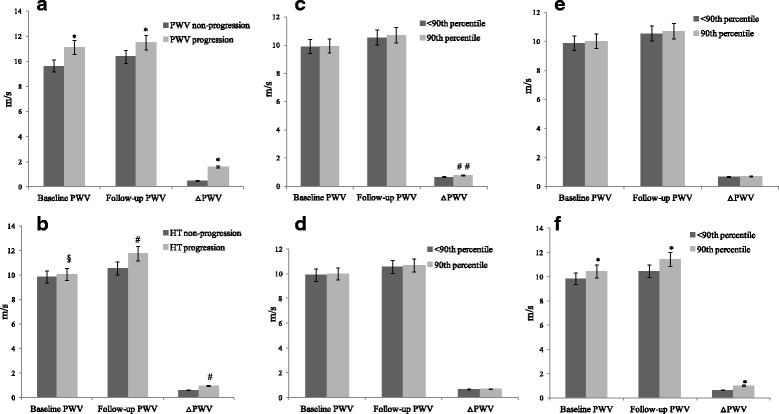
**a** The difference of PWV parameters between PWV progression subjects and non-progression subjects.*p < 0.001. **b** The difference of PWV parameters between hypertension (HT) progression subjects with non-progression subjects. #*p* = 0.024, §*p* = 0.547. **c** Subjects with the 90th percentile SBP had higher △PWV compared with the others. *p* = 0.042.**d** Subject’s PWV parameters were no difference between the 90th percentile and the others according DBP. **e** Subject’s PWV parameters were no difference between the 90th percentile and the others according MAP. **f** Subjects with the 90th percentile age had higher baseline PWV, follow-up PWV and △PWV compared with the others *p < 0.001

We divided all subjects into two groups according to their gender. Male subjects were more likely to smoke and drink than female subjects. However, educational attainment, family income and physical activity were no difference between male and female. Males had significantly higher levels of age(40.5 ± 7.4 vs 37.2 ± 8.1, p < 0.001),BMI (24.8 ± 2.3 vs 21.0 ± 2.2, p < 0.001), blood urea nitrogen (BUN, 5.18 ± 1.23 vs 4.99 ± 1.13, *p* = 0.021), creatinine (Cr, 68.4 ± 10.0 vs 66.7 ± 10.4, *p* = 0.016), sdLDL-C (0.72 ± 0.28 vs 0.66 ± 0.26, *p* = 0.004), sdLDL-C/HDL-C ratio (0.64 ± 0.35 vs 0.58 ± 0.29, *p* = 0.011), baseline PWV (9.96 ± 0.84 vs 9.83 ± 0.85, *p* = 0.03), follow-up PWV (10.66 ± 1.19 vs 10.45 ± 1.18, p = 0.01), △PWV (0.70 ± 0.55 vs 0.62 ± 0.48, p = 0.02) and hs-CRP(0.85 ± 0.21 vs 0.64 ± 0.16, p = 0.004) than females (Table 1). Table 2 showed the difference of PWV parameters, cholesterol components and ratios between PWV, hypertension progression subjects with non-progression subjects. PWV progression subjects had higher levels of baseline PWV, follow-up PWV, △PWV, TG, sdLDL-C and TG/HDL-C ratio. After adjusting for covariates such as age, gender, BMI, FBG, TNF-α, IL-6 and hs-CRP, GLM analyses showed that subjects with PWV and hypertension progression had higher baseline PWV, follow-up PWV and △PWV than those with no PWV and hypertension progression. Meanwhile, GLM analyses also showed subjects with 90th percentile age had higher baseline PWV, follow-up PWV and △PWV compared with the other percentile subjects (*p* < 0.001, Fig. 1).

**Table 1:** Clinical and laboratory data for 816 normotensive subjects divided into two groups according to their gender

Variables	Men (*n* = 476)	Women (*n* = 340)	All subjects (*n* = 816)	*p* value
Age(years)	40.5 ± 7.4	37.2 ± 8.1	39.1 ± 7.8	< 0.001
Education(years)				0.211
Low(<9y)	70(14.7%)	64(18.8%)	134(16.4%)	
Middle(9-12y)	178(37.4%)	120(35.3%)	298(36.5%)	
High(>12y)	228(47.9%)	156(45.9%)	384(47.1%)	
Family income(CNY/y)				0.683
< 30,000	82(17.3%)	59(17.4%)	141(17.3%)	
30,000-100,000	274(57.7%)	187(55.0%)	461(56.6%)	
> 100,000	119(25.0%)	94(27.6%)	213(26.1%)	
Physical activity				0.760
Low	200(42.0%)	155(45.6%)	355(43.5%)	
Intermediate	178(37.4%)	112(32.9%)	290(35.5%)	
High	98(20.6%)	73(21.5%)	171(21.0%)	
BMI(kg/m^2^)	24.8 ± 2.3	21.0 ± 2.2	23.2 ± 2.9	< 0.001
Smoking (%)	163(34.2%)	13(3.8%)	176(21.6%)	< 0.001
Drinking (%)	139(29.2%)	19(5.6%)	158(19.4%)	< 0.001
SBP(mm Hg)	126.2 ± 9.2	125.4 ± 9.6	126.0 ± 9.4	0.245
DBP(mm Hg)	75.0 ± 7.1	75.5 ± 7.1	75.2 ± 7.1	0.344
PP(mm Hg)	51.2 ± 9.5	49.9 ± 9.6	50.7 ± 9.5	0.065
MAP(mm Hg)	92.0 ± 6.5	92.1 ± 6.6	92.0 ± 6.5	0.827
HR(bpm)	72.6 ± 7.2	71.7 ± 7.3	72.2 ± 7.2	0.083
ALT(U/L)	26.5 ± 15.9	28.0 ± 16.4	27.1 ± 16.1	0.196
AST(U/L)	22.8 ± 11.3	22.1 ± 9.5	22.5 ± 10.6	0.391
GGT(U/L)	29.6 ± 18.4	29.5 ± 17.7	29.6 ± 18.1	0.957
BUN(mmol/L)	5.18 ± 1.23	4.99 ± 1.13	5.10 ± 1.19	0.021
Cr(ummol/L)	68.4 ± 10.0	66.7 ± 10.4	67.7 ± 10.2	0.016
UA(ummol/L)	290.8 ± 93.1	291.9 ± 94.8	291.3 ± 93.7	0.874
FBG(mmol/L)	5.11 ± 0.61	5.05 ± 0.61	5.09 ± 0.61	0.186
TC(mmol/L)	4.81 ± 0.77	4.76 ± 0.79	4.79 ± 0.78	0.466
TG(mmol/L)	1.68 ± 0.72	1.62 ± 0.73	1.65 ± 0.73	0.259
LDL-C(mmol/L)	3.00 ± 0.76	2.95 ± 0.77	2.98 ± 0.76	0.349
HDL-C(mmol/L)	1.21 ± 0.30	1.20 ± 0.26	1.21 ± 0.29	0.844
sdLDL-C(mmol/L)	0.72 ± 0.28	0.66 ± 0.26	0.70 ± 0.27	0.004
ApoB(g/L)	0.95 ± 0.26	0.94 ± 0.25	0.94 ± 0.26	0.390
ApoA-1(g/L)	1.21 ± 0.31	1.24 ± 0.30	1.23 ± 0.31	0.102
TC/HDL-C	4.21 ± 1.25	4.14 ± 1.16	4.18 ± 1.21	0.434
TG/HDL-C	1.48 ± 0.79	1.39 ± 0.68	1.44 ± 0.75	0.12
LDL-C/HDL-C	2.64 ± 0.95	2.57 ± 0.90	2.61 ± 0.93	0.312
sdLDL-C/HDL-C	0.64 ± 0.35	0.58 ± 0.29	0.62 ± 0.33	0.011
ApoB/ApoA-1	0.81 ± 0.32	0.82 ± 0.30	0.82 ± 0.31	0.687
Baseline PWV(m/s)	9.96 ± 0.84	9.83 ± 0.85	9.91 ± 0.84	0.03
Follow-up PWV(m/s)	10.66 ± 1.19	10.45 ± 1.18	10.57 ± 1.19	0.01
△PWV(m/s)	0.70 ± 0.55	0.62 ± 0.48	0.67 ± 0.52	0.02
PWV progression (%)	93(19.5%)	51(15.0%)	144(17.6%)	0.094
HT progression (%)	82(17.2%)	47(13.8%)	129(15.8%)	0.189
hs-CRP(mg/L)	0.85 ± 0.21	0.64 ± 0.16	0.76 ± 0.19	0.004
TNF-α(pg/ml)	3.03 ± 0.83	3.04 ± 0.81	3.04 ± 0.82	0.974
IL-6(pg/ml)	2.70 ± 1.17	2.60 ± 1.15	2.66 ± 1.17	0.234

**Table 2:** PWV parameters, cholesterol parameters and ratios and inflammatory factors of subjects by PWV and hypertension progression

Variables	non-PWV progression	PWV progression	*p* value	non-HT progression	HT progression	*p* value
Age(years)	38.5 ± 7.7	41.8 ± 7.7	< 0.001	38.6 ± 8.2	41.7 ± 5.2	< 0.001
Baseline PWV(m/s)	9.63 ± 0.58	11.18 ± 0.71	< 0.001	9.89 ± 0.79	9.99 ± 0.98	0.092
Follow-up PWV(m/s)	10.12 ± 0.67	12.70 ± 0.64	< 0.001	10.46 ± 1.10	11.17 ± 1.43	< 0.001
△PWV(m/s)	0.48 ± 0.27	1.52 ± 0.56	< 0.001	0.63 ± 0.49	0.87 ± 0.62	< 0.001
△SBP(mmHg)	3.11 ± 0.78	3.38 ± 0.85	0.205	2.75 ± 0.74	3.86 ± 0.81	< 0.01
△DBP(mmHg)	2.82 ± 0.69	3.63 ± 0.77	0.039	2.79 ± 0.58	3.81 ± 0.80	< 0.01
△PP(mmHg)	0.26 ± 0.08	0.27 ± 0.06	0.765	0.25 ± 0.09	0.28 ± 0.10	0.113
△MAP(mmHg)	3.08 ± 0.57	3.72 ± 0.61	0.032	2.88 ± 0.45	3.79 ± 0.57	< 0.01
TC(mmol/L)	4.81 ± 0.80	4.69 ± 0.67	0.086	4.80 ± 0.79	4.75 ± 0.75	0.515
TG(mmol/L)	1.58 ± 0.69	1.96 ± 0.83	< 0.001	1.64 ± 0.72	1.72 ± 0.81	0.276
LDL-C(mmol/L)	2.98 ± 0.79	2.96 ± 0.63	0.684	2.99 ± 0.77	2.95 ± 0.70	0.566
HDL-C(mmol/L)	1.20 ± 0.28	1.22 ± 0.29	0.352	1.21 ± 0.28	1.23 ± 0.32	0.469
sdLDL-C(mmol/L)	0.68 ± 0.28	0.77 ± 0.25	< 0.001	0.70 ± 0.27	0.69 ± 0.31	0.661
ApoB(g/L)	0.94 ± 0.25	0.96 ± 0.27	0.301	0.94 ± 0.25	0.96 ± 0.28	0.516
ApoA-1(g/L)	1.23 ± 0.30	1.23 ± 0.33	0.916	1.23 ± 0.30	1.21 ± 0.32	0.523
TC/HDL-C	4.21 ± 1.23	4.02 ± 1.09	0.081	4.19 ± 1.18	4.14 ± 1.37	0.709
TG/HDL-C	1.39 ± 0.72	1.69 ± 0.83	< 0.001	1.43 ± 0.72	1.51 ± 0.89	0.233
LDL-C/HDL-C	2.62 ± 0.95	2.55 ± 0.82	0.398	2.61 ± 0.91	2.59 ± 1.02	0.794
SdLDL-C/HDL-C	0.61 ± 0.34	0.66 ± 0.28	0.063	0.62 ± 0.31	0.61 ± 0.40	0.857
ApoB/ApoA-1	0.81 ± 0.31	0.84 ± 0.32	0.357	0.81 ± 0.31	0.84 ± 0.32	0.338
TNF-α(pg/ml)	3.02 ± 0.82	3.13 ± 0.80	0.153	3.05 ± 0.82	2.99 ± 0.83	0.492
IL-6(pg/ml)	2.69 ± 1.18	2.53 ± 1.12	0.126	2.67 ± 1.17	2.63 ± 1.18	0.738
hs-CRP(mg/L)	0.74 ± 0.19	0.84 ± 0.22	0.298	0.75 ± 0.16	0.84 ± 0.21	0.350

Table 3 gave partial correlation coefficients between PWV parameters and cholesterol components, ratios and inflammation factors after adjustment for age, gender, BMI, exercise, smoking, drinking, SBP, MAP, FBG and UA. Partial Spearman correlation analysis showed that sdLDL-C, TG/HDL-C and IL-6 were correlated weakly but significantly with baseline PWV. sdLDL-C/HDL-C had a weakly correlation with follow-up PWV and △PWV. Both sdLDL-C and TG/HDL-C were significantly correlated with all PWV parameters. Meanwhile, Pearson analysis also confirmed a positive correlation between sdLDL-C and TG/HDL-C (r = 0.433, p < 0.001).

**Table 3:** Partial correlation coefficients between PWV parameters with cholesterol parameters and ratios and inflammatory factors

All subjects (*n* = 816)	Baseline PWV, r(*p*)a	Follow-up PWV, r(*p*)b	△PWV, r(*p*)a
TC	0.022(0.539)	0.038(0.276)	0.051(0.149)
TG	0.019(0.149)	0.022(0.229)	0.029(0.209)
LDL-C	0.001(0.970)	0.004(0.903)	0.007(0.833)
HDL-C	−0.058(0.101)	−0.044(0.212)	− 0.005(0.882)
sdLDL-C	0.064(0.07)	0.113(0.001)	0.149(< 0.001)
ApoB	0.009(0.802)	0.016(0.656)	0.021(0.556)
ApoA-1	−0.058(0.099)	− 0.043(0.219)	− 0.003(0.932)
TC-C/HDL-C	− 0.056(0.112)	− 0.059(0.093)	− 0.042(0.233)
TG/HDL-C	0.092(0.009)	0.152(< 0.001)	0.191(< 0.001)
LDL-C/HDL-C	−0.029(0.406)	−0.022(0.525)	− 0.003(0.933)
sdLDL-C/HDL-C	0.013(0.704)	0.050(0.05)	0.09(0.01)
ApoB/ApoA-1	0.040(0.263)	0.042(0.233)	0.030(0.389)
TNF-α	0.022(0.534)	0.030(0.393)	0.032(0.366)
IL-6	0.090(0.011)	0.076(0.031)	0.025(0.481)
hs-CRP	0.010(0.783)	0.006(0.869)	−0.003(0.942)

Model a was adjusted for gender, age, physical activity, smoking, drinking, BMI, SBP, MAP, FBG and UA

Model b was adjusted for a plus baseline PWV

Then, the associations of sdLDL-C and cholesterol ratios with the PWV parameters were adjusted for covariates using multiple regression models. As shown in Table 4. sdLDL-C was the most closely associated with follow-up PWV and △PWV even after multivariate adjustment. The adjusted regression coefficients (β values) were 0.222 for follow-up PWV, 0.275 for △PWV. However, sdLDL-C and baseline PWV did not show significant association in the multiple regression models (β = 0.192, *p* = 0.065). Homoplastically, sdLDL-C/HDL-C had a similar statistical result to sd LDL-C, accordingly for baseline PWV (β = 0.034, *p* = 0.697), follow-up PWV (β = 0.128, *p* = 0.009) and △PWV (β = 0.137, *p* = 0.011). TG/HDL**-C** was only one cholesterol ratios that associated with all PWV parameters in the multiple regression models.

**Table 4:** Univariable and multivariable linear regression of sdLDL-C and cholesterol ratios with PWV parameters

Variables	Baseline PWV β-Coefficient *p* value		Follow-up PWV β-Coefficient *p* value		△PWV β-Coefficient *p* value	
sdLDL-C						
unadjusted	0.290	0.007	0.623	< 0.001	0.333	< 0.001
adjusted	0.192 a	0.065	0.222 b	< 0.001	0.275 a	< 0.001
TC/HDL-C						
unadjusted	−0.036	0.145	−0.053	0.123	−0.017	0.247
adjusted	−0.044 a	0.061	−0.007 b	0.577	−0.019 a	0.189
TG/HDL-C						
unadjusted	0.126	0.001	0.267	< 0.001	0.141	< 0.001
adjusted	0.095 a	0.012	0.102 b	< 0.001	0.127 a	< 0.001
LDL-C/HDL-C						
unadjusted	−0.025	0.442	−0.026	0.568	−0.001	0.955
adjusted	−0.032 a	0.302	0.005 b	0.753	−0.003 a	0.870
sdLDL-C/HDL-C						
unadjusted	0.100	0.269	0.275	0.031	0.175	0.002
adjusted	0.034 a	0.697	0.128 b	0.009	0.137 a	0.011
ApoB/ApoA-1						
unadjusted	0.070	0.462	0.103	0.439	0.034	0.567
adjusted	0.107 a	0.239	0.022 b	0.667	0.051 a	0.369

Model a was adjusted for gender, age, physical activity, smoking, drinking, BMI, SBP, MAP, FBG, UA, TNF-α, hs-CPR and IL-6

Model b was adjusted for a plus baseline PWV

Further analyses were performed to examine sdLDL-C and cholesterol ratios that were associated with PWV progression. At the end of an average of 5 years of follow-up, 144 subjects had PWV progression. We used the Cox regression model to analyze the predictive value of these cholesterol ratios and sdLDL-C for cf-PWV progression. After adjusted the covariates including gender, age, exercise, smoking, drinking, BMI, SBP, MAP, FBG, UA, baseline PWV, TNF-α, hs-CPR and IL-6, in our study population, sdLDL-C and TG/HDL-C were able to independently predict PWV progression after 5 years (Table 5). It seems that sdLDL-C had higher predicting power than TG/HDL-C (unadjusted: 2.460 vs 1.414; adjusted: 2.070 vs 1.355).

**Table 5:** Cox proportional hazards model for PWV progression^a^

Variables	OR	95%CI	*p* value
sdLDL-C			
unadjusted	2.460	1.400 to 4.321	0.002
adjusted	2.070	1.162 to 3.688	0.014
TC/HDL-C			
unadjusted	0.873	0.754 to 1.012	0.071
adjusted	0.954	0.819 to 1.110	0.541
TG/HDL-C			
unadjusted	1.414	1.185 to 1.687	< 0.001
adjusted	1.355	1.136 to 1.617	0.001
LDL-C/HDL-C			
unadjusted	0.924	0.769 to 1.110	0.396
adjusted	0.896	0.742 to 1.082	0.253
sdLDL-C/HDL-C			
unadjusted	1.379	0.877 to 2.168	0.165
adjusted	1.255	0.780 to 2.020	0.350
ApoB/ApoA-1			
unadjusted	1.178	0.715 to 1.941	0.521
adjusted	1.294	0.786 to 2.129	0.311

^a^ Adjusted for gender, age, physical activity, smoking, drinking, BMI, SBP, MAP, FBG, UA, baseline PWV, TNF-α, hs-CPR and IL-6

**Fig. 2:**
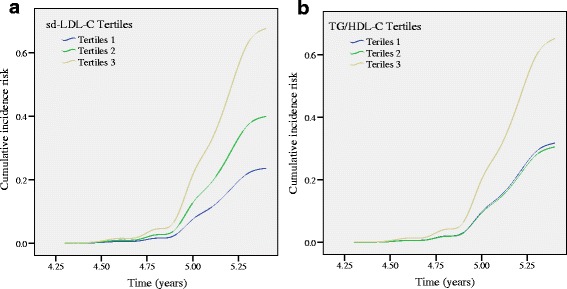
Cox risk regression model analysis showed that the relative risk of arterial stiffness progression in the different tertiles groups of sd-LDL-C and TG/HDL-C ratio

The subjects were grouped based on the tri-sectional quantiles of the sdLDL-C level and TG/HDL-C ratio. TG/HDL-C tri-sectional quantile group was < 1.05, 1.05–1.65, and > 1.65 and sdLDL-C tri-sectional quantile group was ≤0.55 mmol/L, 0.56-0.81 mmol/L and ≥0.82 mmol/L. Cox risk regression model analysis showed that the risk of arterial stiffness progression in the subjects with high sd-LDL-C quantiles were significantly higher than those with low quantiles at the end of the follow-up period (Tertiles 3 vs Tertiles1, RR = 2.867, 95%CI: 1.106 to 7.434, *p* = 0.03). Meanwhile, the risk of high TG/HDL-C ratio quantiles was significantly higher than low quantiles (Tertiles 3 vs Tertiles1, RR = 2.051, 95%CI:1.105 to 3.807, *p* = 0.023). However, there was no significant difference between the sdLDL-C and TG/HDL-C ratio median quantiles and low quantiles subjects with arterial stiffness risk (Fig. 2).

## Discussion

Based on the Framingham Heart Study published in 1959, cholesterol levels were the first confirmed cardiovascular risk factor [21]. Currently, it is understood that blood lipid parameters and early atherosclerosis [22], CIMT [12, 14] and arterial stiffness [23] are connected. sdLDL-C was considered as the best marker for predicting carotid atherosclerosis [11]. However, the association between sdLDL-C and arterial stiffness was still unclear. We systematically and simultaneously evaluate the effects of sdLDL-C and cholesterol ratios on arterial stiffness progression in normal blood pressure subjects. With an average of 5 years follow-up, we found that sdLDL-C and TG/HDL-C ratio could independently predict arterial stiffness progression after adjusting for other cardiovascular risk factors. Subjects with a high sdLDL-C level and TG/HDL-C ratio had a higher risk of arterial stiffness.

Previous studies showed that sdLDL-C was the best marker for predicting carotid arteriosclerosis by using CAIMT [11]. sdLDL-C was a quantitative risk marker of arteriosclerosis than the standard lipid parameters [10]. The key finding of the present study was further confirmed that sdLDL-C was a better lipid variable than other cholesterol ratios in assessing the risk of arterial stiffness progression using cf-PWV. sdLDL-C may be the best indicator for predicting arterial stiffness in all lipid parameters and cholesterol ratios. There are several explanations for this finding. First, as compared with the larger size counterparts, sdLDL-C particles have lower affinity to LDL receptors [24], and have longer residence time in the circulation [25]. Second, due to sdLDL-C particles are small and have larger specific surface area, they are easier penetration into arterial wall [26]. Third, sdLDL-C is one of the most atherogenic lipoprotein classes and higher susceptibility to oxidative modification [27]. Finally, sdLDL-C level may be an integrated marker for atherogenic risk [10, 28]. sdLDL-C was strongly correlated with TG, HDL-C and apoA-1 [11]. In our healthy population, the baseline sdLDL-C levels of PWV progression subjects were significantly higher than non progressive subjects. sdLDL-C can predict progression of arterial stiffness progression independently with other cardiovascular risk factors. However, sdLDL-C levels were not associated with baseline PWV. These results show that sdLDL-C gradually plays a role in promoting arterial stiffness progression.

In our normal blood pressure population, high TG/HDL-C ratio subjects have a higher risk of arterial stiffness. This may be associated with the fact that TG/HDL-C can better predict sdLDL-C, which is a kind of atherosclerotic lipid particle that can strongly predict CVD [10]. Under the effect of cholesterol ester transfer protein, higher TG levels correlate with more active lipid exchanges, resulting in increased sdLDL-C concentrations and decreased HDL-C levels, which ultimately enhance arteriosclerosis-inducing factors [29]. King et al. indicated that when the cut-off point for TG/HDL-C was 1.4, specificity and sensitivity reached 79% and 80%, respectively, when using this index to predict sdLDL-C, and thus it is the best known predicator [30]. In our population, the average TG/HDL-C ratio was 1.44 ± 0.75, whereas the ratio of PWV progression subjects was significantly higher than non-progression (1.69 ± 0.83 vs 1.39 ± 0.72, *p* < 0.001). Meanwhile, TG/HDL-C ratio and sdLDL-C also showed a significant positive correlation. Interestingly, TG/HDL-C was associated with baseline PWV, whereas sdLDL-C and sdLDL-C/HDL-C were not correlated with it.

Previously, a limited number of studies have addressed the relation between ApoB and arterial PWV. In relatively small study populations, using multiple linear logistic regression models found that ApoB and PWV had independent positive associations after adjusting for cardiovascular risk factors [31]. In addition, ApoB and the ApoB/ApoA-1 ratio were found to predict PWV assessed 6 years later [32]. However, in our population, ApoB/ApoA-1 ratio has no association with PWV change and the follow-up PWV after 5 years. ApoB levels reflect the total number of atherogenic particles (very lowdensity lipoprotein [VLDL], VLDL remnants, LDL, lipoprotein [a]). However, it is influenced by genes and its level has an obvious difference in various ethnic groups [33]. For instance, ApoB levels in China’s healthy population are significantly lower than in India. PWV is also affected by genes [34].

Many studies have suggested that arteriosclerosis was an inflammatory reaction secondary to vascular injury, and inflammatory factors have a sustained effect on whole process of arteriosclerosis. In healthy individuals, acute systemic inflammation increases arterial stiffness and decreases wave reflections [35]. Azra Mahmud also founded that arterial stiffness is related to systemic inflammation including TNF-α, IL-6 and hs-CRP in essential hypertension [36]. We measured TNF-α, IL-6 and hs-CRP levels in our healthy subjects at baseline. After adjusted cardiovascular risk factors, partial Spearman correlation analysis showed that only IL-6 was correlated weakly with baseline and follow-up PWV. Both sdLDL-C and TG/HDL-C are able to independently predict arterial stiffness progression after adjusted cardiovascular risk factors and inflammatory factors. High level of sdLDL-C and disproportion of TG and HDL-C may be an initiating factor to promote arterial stiffness progression in healthy.

This study has several limitations. Dyslipidemia in the Chinese population is mainly type IV hyperlipidemia, characterized by hypertriglyceridemia and low HDL-C, which is closely related to the carbohydrate-based diet in the general Chinese population [37]. Additionally, more subjects included in the study accepted tests in the cold season, and the temperature change may have affected the dietary structure of the population, resulting in increased variability of lipid components. Although this study is a multicenter study, however, it is not a multi-ethnic study. It is important to clarify that genes have an effect on PWV and sdLDL-C.

## Conclusions

In conclusion, our study showed that sdLDL-C and TG/HDL-C ratio could independently predict arterial stiffness progression after adjusting for other CVD risk factors. Subjects with a high sdLDL-C level and TG/HDL-C ratio had a higher risk of arterial stiffness. However, it remains to be confirmed whether these conclusions can be applied to other populations.

## Abbreviations

△PWV: Change from baseline PWV to follow-up PWV
ALT: Alanine aminotransferase
Apo B: Apolipoproteins B
ApoA-1: Apolipoproteins A-1
AST: Aspartate aminotransferase
BMI: Body mass index
BUN: Blood urea nitrogen
CAVI: Cardio-ankle vascular index
cf-PWV: Carotid-femoral pulse wave velocity
CIMT: Carotid intima media thickness
Cr: Creatinine
CVD: Cardiovascular disease
DBP: Diastolic blood pressure
FBG: Fasting blood glucose
GGT: Gamma-glutamyltransferase
HDL-C: High density lipoprotein cholesterol
HR: Heart rate
hs-CRP: High-sensitivity C reactive protein
HT: Hypertension
IL-6: Interleukin-6
LDL-C: Low-density lipoprotein cholesterol
MAP: Mean arterial pressure
PP: Pulse pressure
SBP: Systolic blood pressure
sdLDL-C: Small dense low-density lipoprotein cholesterol
TC: Total cholesterol
TNF-α: Tumor necrosis factor α
UA: Uric acid
